# MitoTimer: a novel protein for monitoring mitochondrial turnover in the heart

**DOI:** 10.1007/s00109-014-1230-6

**Published:** 2014-12-06

**Authors:** Roberta A. Gottlieb, Aleksandr Stotland

**Affiliations:** Heart Institute and Barbra Streisand Women’s Heart Center, Cedars-Sinai Medical Center, 127 S. San Vicente Blvd. AHSP9105, Los Angeles, CA 90048 USA

**Keywords:** MitoTimer, Mitochondrial biogenesis, Mitophagy, Fluorescence microscopy

## Abstract

Mitochondrial quality control refers to the coordinated cellular systems involved in maintaining a population of healthy mitochondria. In addition to mitochondrial protein chaperones (Hsp10, Hsp60, and others) and proteases (Lon, AAA proteases) needed for refolding or degrading individual proteins, mitochondrial integrity is maintained through the regulation of protein import via the TOM/TIM complex and protein redistribution across the network via fusion and fission and through mitophagy and biogenesis, key determinants of mitochondrial turnover. A growing number of studies point to the importance of mitochondrial dynamics (fusion/fission) and mitochondrial autophagy in the heart. Mitochondrial biogenesis must keep pace with mitophagy in order to maintain a stable number of mitochondria. In this review, we will discuss the use of MitoTimer as a tool to monitor mitochondrial turnover.

## Mitochondrial turnover

Mitochondria have tissue-specific rates of turnover. For example, in the mouse heart, mitochondria turn over with a half-life of 14 days [1], but in the liver, the half-life is 2–4 days [2, 3]. Selective mitochondrial autophagy, or mitophagy, eliminates damaged and dysfunctional mitochondria [4] and is closely linked to mitochondrial biogenesis, which permits replacement of mitochondria (or synthesis of components and their insertion into the remaining functional mitochondria). Rat cardiomyocytes have ~10,000 mitochondria per cell [5–9]; this suggests that under resting conditions, one mitochondrial unit per cell is replaced every 4 min (assuming turnover proceeds at a constant rate). Mitochondria comprise one third of the volume of the heart and under normal conditions are responsible for 93 % of ATP production (glycolysis is responsible for 3–7 %, but this may rise considerably during ischemia) [10]. However, mitochondria are not producing ATP at maximal rates all of the time, rather they are heterogeneous [11]: some mitochondria may be producing ATP at high rates while others may be relatively inefficient or quiescent or producing significant amounts of reactive oxygen species (ROS). Mitochondria routinely sustain oxidative damage as a by-product of oxidative phosphorylation. However, this is ordinarily handled by targeted protein quality control (Lon protease and other AAA proteases) [12] and by exchange of components during fusion followed by fission [1, 11]. Asymmetric fission allows for segregation of dysfunctional components into one daughter mitochondrion that can be removed by autophagy [11]. A number of enzymes involved in oxidative phosphorylation (OX-PHOS) are expressed with diurnal variation [13], suggesting that mitochondrial regeneration is regulated by the circadian cycle, and further implies that mitochondrial elimination might be similarly regulated. This seems likely given that a period of fasting occurs during sleep; therefore, waves of mitochondrial elimination may alternate with regeneration on a daily basis, with 3–4 % of the mitochondrial mass (or population) being replaced each day [3]. However, this may be much higher: in HL-1 cells subjected to serum and amino acid starvation (glucose present) for 3.5 h, the number of mitochondria is reduced by 70 % [14].

## Benefits of mitophagy

Previous work by our lab has demonstrated that Parkin-dependent mitophagy is essential for the protective effect of ischemic preconditioning [15]. The selection of mitochondria for elimination by autophagy is related to low membrane potential and possibly other factors such as ROS production [16]. Since mitochondria with low membrane potential will not produce much ATP, their selective elimination may not have a noticeable impact on cellular ATP levels. In fact, elimination of depolarized mitochondria could increase cellular ATP by reducing the futile hydrolysis of ATP in an effort to restore mitochondrial membrane potential. Furthermore, eliminating dysfunctional mitochondria might attenuate ROS production, and the remaining mitochondrial population might exhibit better calcium homeostasis and greater resistance to MPTP induction. In rats subjected to 50 % caloric restriction for 1 week followed by 1 week of refeeding, the hepatic mitochondria were shown to be more efficient, as reflected by higher state 3 mitochondrial respiratory capacity and increased superoxide dismutase activity [17].

Failure of mitochondrial quality control is accompanied by progressively lower membrane potential, decreasing cellular ATP levels, and increased ROS production [18]. Complex I and aldehyde dehydrogenase 2 (ALDH2) are well known to be vulnerable to oxidative damage, and specific aldehyde modifications are detected in mitochondria from hearts after acute oxidative stress [19, 20]. Cardioprotection reduces the abundance of these modifications, and we suggest this is mediated in part by mitophagy and biogenesis.

## Mitophagy is balanced by biogenesis

In order to maintain cellular homeostasis, elimination of mitochondria must be balanced by their replacement with newer, more efficient mitochondria. The transcriptional co-activator peroxisome proliferator-activated receptor gamma co-activator 1-alpha (PGC-1α) is the master regulator of mitochondrial biogenesis. Interestingly, many of the same regulatory elements found in the promoters of OX-PHOS genes are also found in the promoters of autophagy genes, suggesting coordinate regulation (L van der Stap, KD Finley, and RA Gottlieb, unpublished data). PGC-1α also regulates expression of TFEB, the key transcriptional regulator of autophagy and lysosomal biogenesis. The peroxisome proliferator-activated receptor gamma (PPARγ) in tandem with the retinoic acid receptor (RXR) regulates pathways of fatty acid oxidation for both mitochondria and peroxisomes. Free fatty acids, clofibrate, and thiazolidinediones are ligands for PPARγ and stimulate mitochondrial biogenesis. Mitochondrial biogenesis is controlled by the PPARγ coactivator (PGC) family of transcriptional coactivators, most importantly PGC-1α, PGC-1β, and the PGC-related coactivator PRC. PGC-1α works in tandem with nuclear respiratory factor 2 (NRF-2) to co-activate NRF-1 [21]. The NRFs direct the transcription of nuclear-encoded mitochondrial proteins, the mitochondrial protein import machinery, and co-factors required for assembly of the respiratory chain complexes, as well as the regulatory factors required for mitochondrial DNA transcription and translation, most importantly mitochondrial transcription factor A (Tfam). Tfam is important not only for mitochondrial gene transcription but also for maintenance of mitochondrial DNA (mtDNA) copy number [22]. Overexpression of PGC-1α is sufficient to drive mitochondrial biogenesis [23]. Acetylation of PGC-1α suppresses its transcriptional co-activator activity and can therefore limit mitochondrial biogenesis despite high levels of protein. Therefore, an important regulator of mitochondrial biogenesis is the histone deacetylase sirtuin 1 (Sirt1) which can serve to activate autophagy as well as PGC-1α and mitochondrial biogenesis [24, 25]. Another linkage between mitophagy and biogenesis is PARIS (also known as ZNF746), a substrate of Parkin that functions as a transcriptional repressor of PGC-1α [26].

## Additional pathways converge on mitochondrial quality control

Mitochondria possess machinery for responding to an imbalance between imported nuclear-encoded proteins and mitochondrial-encoded proteins, known as the mitochondrial unfolded protein response (UPRmt). The correct stoichiometry of OX-PHOS subunits is essential for assembly of functional respiratory complexes. Excess or unincorporated subunits are degraded to short peptides by matrix proteases of the AAA+ family (ATPases associated with a variety of cellular activities); Lon protease is responsible for the largest share of intramitochondrial protein degradation. In *Caenorhabditis elegans*, the peptide fragments generated by the related protease ClpP are extruded into the cytosol through the transporter Haf1 [27]. The mammalian homolog is thought to be ABCme10, and the extruded peptides are recognized by transcription factors CHOP, C/EBPβ, and cJun/AP1, which direct the expression of mitochondrial chaperone hsp60 and proteases ClpP, Yme1L1, and MPPβ. Upregulation of these factors reduces protein aggregation in the mitochondrial matrix. Additional proteins upregulated in the UPRmt include Tim17A, NDUFB2, and EndoG, but not ER stress proteins Bip, Grp94, calreticulin, or calnexin [28]. PKR (double-stranded RNA-activated protein kinase) phosphorylates eIF2α and cJun, with the resulting suppression of translation and import of nuclear-encoded mitochondrial proteins [29]. There is almost nothing known about the significance of the UPRmt in the mammalian heart or its connection to mitophagy and mitochondrial biogenesis. However, agents such as chloramphenicol and rapamycin, which induce mitophagy/autophagy, are also reported to be potent inducers of UPRmt [30]. How the UPRmt may affect MitoTimer protein turnover and whether the overexpression of MitoTimer affects the UPRmt are unknown but may need to be considered when interpreting results.

## Fluorescence properties of MitoTimer

**Fig. 1:**
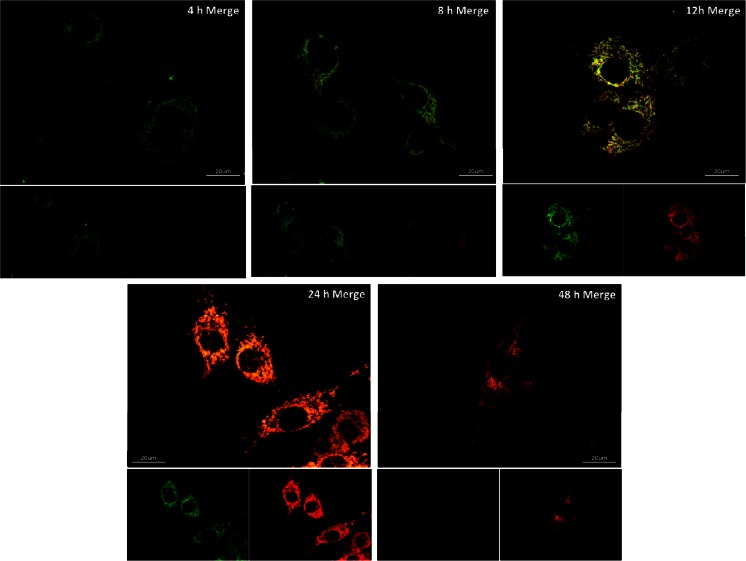
Maturation of MitoTimer in C2C12 cells. Cells stably transfected with rtTA and pTRE-tight-MitoTimer were exposed to doxycycline for 1 h followed by media exchange, then imaged at the indicated times. Each *large panel* shows the merged image while the insets show the individual *red* and *green channels*. Synchronized maturation of MitoTimer is evident

**Fig. 2:**
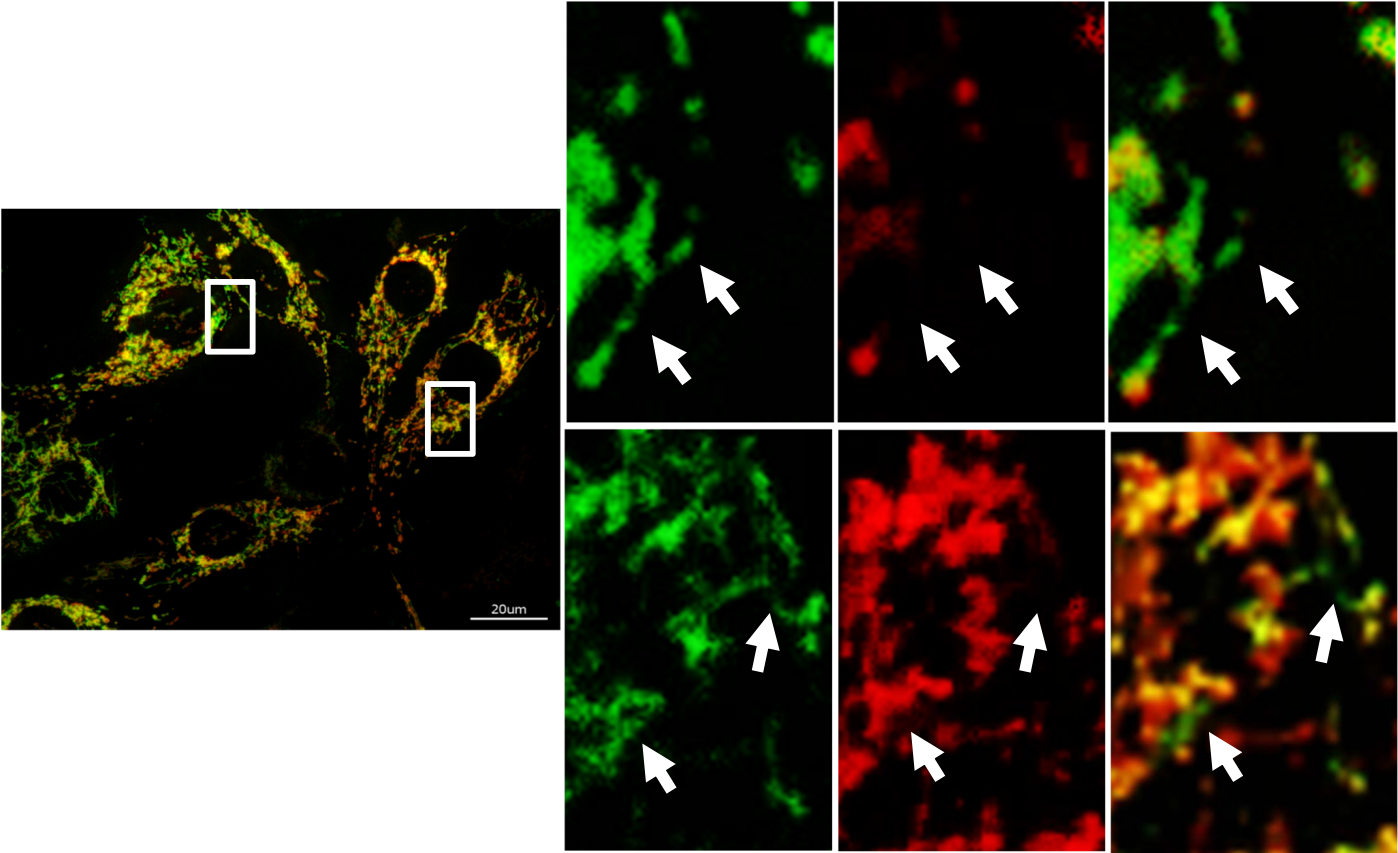
Protein import in the mitochondrial network is spatially inhomogeneous. A field of C2C12 cells exposed to doxycycline continuously for 24 h is shown. *Rectangles* outline areas that are enlarged at *right*; *arrows* indicate regions of the mitochondrial network which contain *green* MitoTimer (newly synthesized and imported) but lack mature (*red*) MitoTimer

**Fig. 3:**
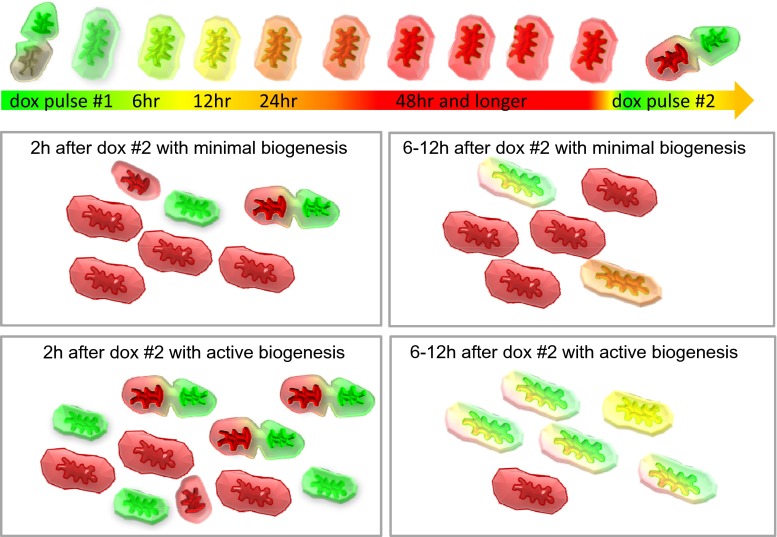
Schematic of two-pulse Dox induction of MitoTimer. Dox is added to media for 2 h, then washed out, and MitoTimer is allowed to mature for 48 h, after which a second pulse of Dox is given in the presence or absence of an agent to stimulate mitochondrial biogenesis. Cells are imaged a few hours later. Under conditions of active mitochondrial biogenesis, more mitochondria have imported new (*green*) MitoTimer than in unstimulated cells, where there is minimal biogenesis and correspondingly little import of newly synthesized MitoTimer protein

Recent advances in assessing mitochondrial turnover include the analysis of the half-lives of mitochondrial proteins using mass spectrometry analysis and deuterium labeling [3], mito-Keima, which can report on mitochondria delivered to the lysosome [31], and MitoTimer, a fluorescent protein that can be used to monitor mitochondrial turnover [32–34]. Kim et al. demonstrated that deuterium labeling coupled with gas chromatography-mass spectrometry can reveal differential turnover rates of mitochondrial proteins on a proteome-wide level [3]. This level of analysis of mitochondrial turnover rate is highly informative in whole organs but is not suitable for analysis of mitochondrial turnover on a level of discrete mitochondria. Likewise, while mito-Keima is a useful tool in quantitative assessment of single mitophagic events and allows for monitoring the rate of mitophagy in cells, it does not allow for monitoring of mitochondrial biogenesis. The major advantage provided by MitoTimer is that it allows for the monitoring of mitochondrial turnover and protein import (with resolution ranging from whole tissues down to individual mitochondria). Timer is a fluorescent protein developed by Terskikh as a mutant of DsRed [35]. Timer molecules transition from green fluorescence to a more stable red conformation over a span of 48 h [Fig. 1]. Timer or fusion protein derivatives have been used to monitor various processes including gene expression, intracellular protein recycling, cell survival, and the kinetics of viral infection [36–39]. For the purpose of tracking mitochondrial turnover, a useful approach is to express MitoTimer under the control of a tetracycline response element (TRE) such as pTRE-tight [33] in conjunction with the reverse tetracycline transactivator (rtTA). Synchronized expression of MitoTimer is achieved by inducing expression for a brief period of time with addition of doxycycline (Dox) pulse, followed by removal of any residual Dox (chase). The protein can be monitored as it matures from green to red over 48 h, and it is retained in the mitochondria for a number of days (presumably corresponding to a mitochondrial half-life). A second Dox pulse can be used to induce another round of expression, which is readily evident as green MitoTimer [Fig. 2 and schematic shown in Fig. 3]. MitoTimer matures within 48 h, a rate which parallels mitochondrial turnover in the liver, but considerably faster than turnover rates in the heart. Thus in tissues with slow mitochondrial turnover, the presence of red mitochondria after a brief induction with doxycycline cannot allow one to distinguish between mitochondria that imported MitoTimer 2 days ago and mitochondria that imported the protein 1 or 2 weeks ago. To examine mitophagy, one must monitor the rate of disappearance of (red) MitoTimer, but in dividing cells (even stably transfected cells), one cannot distinguish between dilution of the existing fluorescent protein due to cell division and loss of protein through mitophagy. Thus monitoring basal rates of mitochondrial turnover in immortalized cells may be difficult. However, MitoTimer can be used to monitor mitophagy that is acutely upregulated in response to a stressor. Similar challenges and limitations exist with monitoring mitochondrial biogenesis, but these can be partially overcome by using a second pulse of doxycycline before or during the intervention that is proposed to induce biogenesis. A diagram of the typical two-pulse labeling protocol is shown in Fig. 3. While a second doxycycline addition triggers a rather small amount of MitoTimer incorporation under basal conditions (probably 3–4 %, corresponding to the net turnover rate), an intervention that triggers biogenesis will result in a much larger incorporation of newly synthesized MitoTimer. Given that MitoTimer is under the control of the artificial TRE-tight promoter, the differential protein translation and import under basal and stimulated conditions is likely due to posttranscriptional control through the native cox8 mitochondrial targeting sequence added to Timer [40]. Finally, it is possible to gain insight into the relative equilibrium of mitophagy and biogenesis under conditions of constitutively expressed MitoTimer, where a change in the red to green ratio reflects a shift in the equilibrium.

## Specific considerations and protocols for using MitoTimer

When utilizing MitoTimer to monitor mitochondrial turnover, several factors need to be taken into account. Timer maturation rate has previously been shown as independent of protein concentration, ionic strength, and a wide range of biological pH values [35]. In our studies with MitoTimer, we have found that the maturation rate was, however, accelerated during live fluorescence imaging of the cells. When fixed in 4 % paraformaldehyde, MitoTimer retains the conformation it was in at the time of fixation, thus permitting prolonged imaging of the cells. Furthermore, depending on the fluorescent microscope used in the experiments, filter sets may need to be optimized in order to detect the green signal of MitoTimer. For example, in our previous studies, we utilized a Nikon (TE300, Nikon, Melville, NY) microscope equipped with a cooled CCD camera (Orca-ER, Hamamatsu, Bridgewater, NJ) and automated excitation emission filter wheels controlled by a Lambda 10–2 (Sutter instruments) operated by MetaMorph Version 6.2r (Molecular Devices); in order to maximize the MitoTimer signal, we used a triple-cube Chroma 61002-triple emitter 61002, with the added green emission filter Chroma 233755 D520–40 m (midpoint 520, emission 500–540) and the red emission filter Chroma 221379 D605–55 (midpoint 605, emission 578–632). It is also important to make sure that threshold levels used in collecting the images from MitoTimer cells remain the same throughout the time course experiment. Inducible expression of MitoTimer is a useful approach in experiments utilizing cell lines, as time-restricted induction can provide insights into mitochondrial dynamics and subpopulation. Inducible expression in a stably transfected cell line also avoids the effect of leaky plasmid promoters, heterogeneous protein expression intrinsic to transient transfection, and the abnormal accumulation of messenger RNA (mRNA), all of which may mask the visualization of MitoTimer maturation in discrete mitochondria. Constitutive expression of MitoTimer can, however, be informative in in vivo experiments, as demonstrated by Laker et al. [32]. In this context, mitochondrial dynamics and import rates are much more heterogeneous than those observed in immortalized tumor-derived cell lines (especially if expressed under tissue-specific promoters), and a shift in the abundance of newly expressed vs. mature MitoTimer can be used to reveal changes in the rate of mitochondrial turnover (the balance of biogenesis and mitophagy). In our work, we utilized the well-characterized tetracycline (Tet)-On inducible expression system with a brief doxycycline (Dox) pulse in order to synchronize the uptake of newly synthesized MitoTimer by the mitochondria. Although this system allows for conditional expression, it still has the drawback of requiring transient transfection with two plasmids and can be unwieldy given the need to transfect, induce expression, and monitor maturation of MitoTimer during a particular intervention. To solve this problem, we found that the best level of control in the system could be achieved by using retroviral transfer vectors to express the reverse Tet transactivator and MitoTimer under the control of the Tet responsive elements, which we used to establish stable cell lines derived from the C2C12 skeletal muscle myoblast cell line and the murine atrial cardiomyocyte HL-1 cell line. We further improved the control of the system using fluorescence-activated cell sorting to sort first for cells that were negative for MitoTimer in the absence of Dox; the sorted cells were then stimulated with Dox followed by a second round of sorting to select cells with the highest expression of MitoTimer. The resulting cell lines exhibited tight control of MitoTimer expression and required only a brief (2 h) pulse with 2 mg/ml Dox in order to achieve a high level of expression. Following the pulse, it is important to completely remove Dox from the media by washing several times with phosphate-buffered saline and replacing with fresh media (and tetracycline-free serum), as even a trivial concentration of Dox is sufficient to induce expression in cell lines selected for high responsiveness. Utilizing the method of cell line selection described above, we have been able to express MitoTimer in an inducible fashion across a wide range of cell lines; this approach is strongly recommended for in vitro studies. MitoTimer is also a tool highly suitable for analyzing and sorting mitochondria by flow cytometry. When used for this purpose, it is highly recommended that single positive green fluorescent and red fluorescent cells or mitochondria be used in setting up the parameters of the sort to compensate for the bleed-through of green fluorescence into the red channel. This step is crucial in obtaining an accurate picture of the levels of green-to-red fluorescence of individual cells and mitochondria and should be performed alongside the MitoTimer experiments as part of the controls.

## Use of MitoTimer to investigate mitochondrial biology

We targeted Timer to the mitochondrial matrix (MitoTimer). The rate of diffusional exchange of contents across the mitochondrial network is fastest for outer membrane constituents, intermediate for matrix contents, and slowest for inner membrane proteins [18]. Mitochondrial fusion events require high membrane potential [11] and mitofusin-1 or mitofusin-2 (Mfn1/2) [34]. In cells with a normal mitochondrial network, MitoTimer of varying degrees of maturation (green to red) is distributed homogeneously across the network. However, in mouse embryonic fibroblasts (MEFs) derived from Mfn1/2 double knockout mice, distribution of new vs. mature MitoTimer was heterogeneous [34], demonstrating that fusion events enable mixing of old and new proteins within the mitochondrial network. Neurons also exhibit heterogeneous distribution of MitoTimer, with newly synthesized protein being incorporated into mitochondria closest to the soma while mature MitoTimer predominated in the distal portion of the neurites. In fact, the red to green ratio was proportional to distance from the soma [34]. Interestingly, it has been reported that autophagosomes initiate distally in neurons (near the oldest mitochondria) and then migrate towards the soma, where they fuse with lysosomes [41]. These examples illustrate the use of MitoTimer to gain spatiotemporal information about mitochondrial age.

## Use of MitoTimer to assess protein import

MitoTimer also provides information about the level of mitochondrial protein import across a population of cells and organelles. High membrane potential is required for mitochondrial protein import [42]. Mitochondria which lose membrane potential will be unable to import new (green) MitoTimer protein. If biogenesis is impaired, one would expect a decrease in the number of mitochondria importing new protein, which would be reflected by a decrease in green MitoTimer in mitochondria. Conversely, increased biogenesis would be reflected in an increase in green MitoTimer in mitochondria. This was demonstrated in our study in which we showed that cells recovering from FCCP or statin treatment showed an increase in new MitoTimer protein import (green mitochondria) [33] during a second period of doxycycline induction (using a tetracycline-inducible construct and rtTA-expressing cells). Using constitutive expression of MitoTimer in a *Drosophila* heart tube, Laker et al. showed that exercise training resulted in an increase in the abundance of green mitochondria [32], consistent with increased mitochondrial biogenesis. Oddly, the authors attributed this green predominance to decreased oxidative stress in the exercised mice, although it has been shown that exercise increases reactive oxygen species in skeletal muscle mitochondria [43], and MitoTimer color maturation was shown to be insensitive to reactive oxygen species [34]. Interestingly, MitoTimer import during biogenesis is not uniform but is enriched in a perinuclear zone in C2C12 cells [Fig. 2] [33] and in neuronal cells [34]. While this may be simply due to mRNA proximity, it is possible that the subpopulation of mitochondria enriched for green MitoTimer is specialized for mitochondrial regeneration.

## Use of MitoTimer to assess mitochondrial turnover

Mitochondrial turnover also depends upon clearance of mitochondria by mitochondrial autophagy or mitophagy. Suppression of autophagy results in the accumulation of mitochondria with lower membrane potential and increased oxidative damage [44]. In autophagy-deficient cells in which MitoTimer expression was induced for 4 h and allowed to mature for 48 h, there was increased accumulation of red MitoTimer compared to autophagy-competent cells [34], indicating that mitochondrial turnover was impaired. Similarly, in mice in which MitoTimer was constitutively expressed in skeletal muscle (after electroporation of virus), the ratio of red to green MitoTimer was increased in mice on a high-fat diet compared to normal chow-fed animals; the ratio was decreased in mice subjected to an exercise regimen compared to sedentary mice on the same diet [32]. Collectively, these studies show that MitoTimer can be used to assess mitochondrial turnover. Analysis of mitochondrial turnover will contribute to our understanding of disease as acquired mitochondrial dysfunction can be compensated for through mitophagy and biogenesis. Loss of this compensatory process results in the accumulation of damaged mitochondria which may generate increased reactive oxygen species; consume ATP through reversal of the ATP synthase; disrupt calcium homeostasis; activate innate immunity, leading to inflammation; or release cytochrome c, thereby triggering cell death.

## Caveats and limitations of MitoTimer

The use of MitoTimer to date has been limited to cell culture, with limited studies in flies and electroporation into mouse skeletal muscle. However, the findings have engendered considerable enthusiasm for the technology, and the pTRE-tight-MitoTimer plasmid has been requested by 18 groups since it was deposited with Addgene in April. There are some caveats to consider when using MitoTimer, however. Given the wide range of turnover of different proteins in mitochondria [3], it is important to confirm that MitoTimer turnover is reflecting mitochondrial turnover. This can be done by testing interventions that either inhibit or accelerate mitophagy to confirm that MitoTimer clearance changes accordingly. Similar verification is needed for estimates of biogenesis. Unfortunately, the chemical treatment used for 5-ethynyl-2′-deoxyuridine (EdU) staining destroys fluorescent proteins, making the two mutually incompatible, although paired samples can be stained. Nevertheless, if both protocols show the same trend, then one can feel assured that MitoTimer is behaving as one might expect, with respect to biogenesis. The fluorescent protein is subject to photoconversion (green to red) by persistent light exposure, so time-lapse imaging may not be feasible. Use in primary cells depends upon having the appropriate viral vectors for efficient gene delivery, and establishing transgenic mice will be invaluable.

## Comparison of methods to monitor mitophagy

Currently three recently introduced methods provide information on mitochondrial turnover. The use of deuterium labeling and mitochondrial proteome analysis is extremely powerful and can be used in cells, non-transgenic animals, and humans [3]. However, it provides limited subcellular information (although the interfibrillar and subsarcolemmal mitochondrial proteomes could be measured separately) and requires sophisticated mass spectrometry and bioinformatics capability. Mito-Keima is an elegant reporter for mitophagy [31] that will become extremely useful once transgenic lines are available. Its chief limitation is that it can only report on the final phase of mitophagy (delivery to the lysosome) and is only applicable for fluorescence microscopy. MitoTimer provides information on biogenesis and, by using time-restricted expression, can also provide information on mitophagy [33]. Constitutive expression of MitoTimer can provide information on the relative balance of mitophagy and biogenesis [32, 34]. Currently the approach is limited to cell culture or electroporation into tissues; however, adenoviral delivery to the heart is feasible, as is production of a transgenic mouse with either the tetracycline-inducible promoter or a constitutive promoter. Mitochondrial biogenesis can also be monitored in cells or in vivo by incorporation of tagged nucleotides such as BrdU or EdU into mitochondrial DNA; successful labeling of mtDNA was described by Oka et al. [45].

## Conclusions

Impaired mitochondrial quality control is frequently a consequence of impaired autophagy in conditions such as advanced age or the metabolic syndrome. Loss of mitochondrial quality control may contribute to many chronic disease phenotypes. The use of MitoTimer to monitor mitochondrial turnover may reveal new insights into mitochondrial biology in health and disease.
